# Supplementary figures and images for: Adrenalectomy for Metastasis: The Impact of Primary Histology on Survival Outcome
Source: Cancers (Basel). 2024 Feb 13;16(4):763. doi: 10.3390/cancers16040763 (PMC10886600; doi:10.3390/cancers16040763)

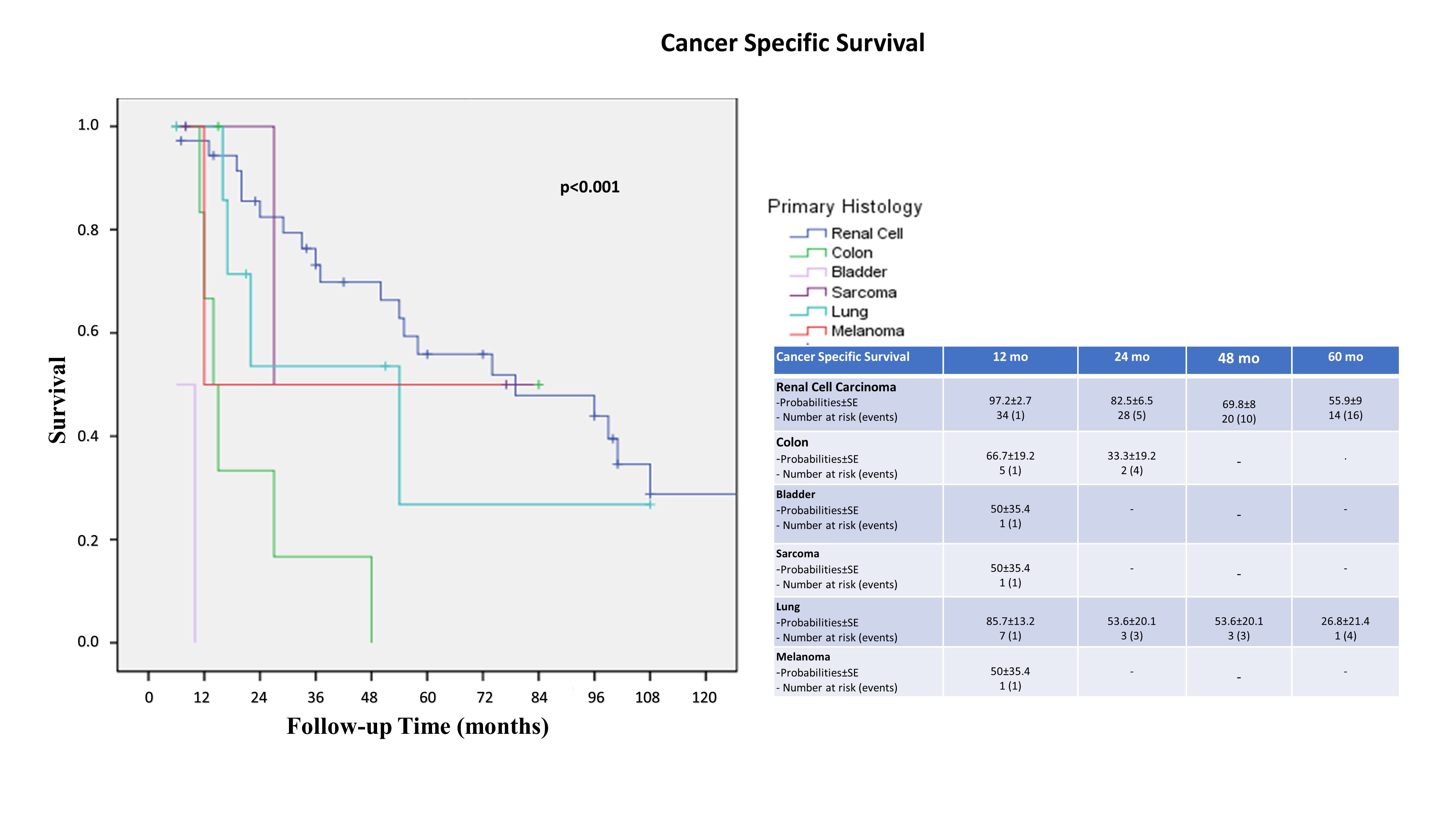

Supplement: Supplementary file 1 [file cancers-16-00763-s001.zip › cancers-2802918-supplementary.jpg]
